# Adult megakaryopoiesis: when taking a short-cut results in a different final destination

**DOI:** 10.1097/BS9.0000000000000202

**Published:** 2024-08-15

**Authors:** Wenxu Zhu, Gavin Tjin, Louise E. Purton

**Affiliations:** a Stem Cell Regulation Unit, St. Vincent’s Institute of Medical Research, Fitzroy, VIC 3065, Australia; b The University of Melbourne Department of Medicine at St. Vincent’s Hospital, Fitzroy, VIC 3065, Australia

It is now well-established that there are 2 distinct pathways for megakaryopoiesis. One is defined by the traditional stepwise differentiation pathway that progresses from hematopoietic stem and progenitor cells (HSPCs) to common myeloid progenitors (CMPs), megakaryocyte-erythroid progenitors (MEPs), megakaryocyte progenitors (MkPs), and then to megakaryocytes (MKs), which give rise to platelets.^1,2^ The other pathway has more recently been reported as the direct differentiation pathway from HSPCs to MkPs^3,4^ (**Fig. 1A**). Recent studies by Li et al^5^ have revealed that the 2 pathways respond differently to inflammation and myeloablation stressors, producing inflammatory or niche-supporting MKs, respectively, in addition to platelet-producing MKs.^5^

**Figure 1.**
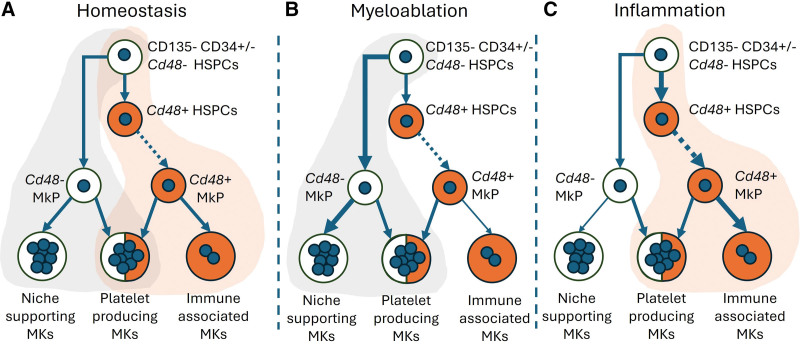
Differential MkP/megakaryocyte maturation pathways produce functionally distinct megakaryocytes. (A) The *Cd48*^dre^;*R26*^rox-tdTomato^ reporter mouse model differentiates between MkPs and MKs that are formed from direct differentiation (unlabeled; white) and those produced through the classical stepwise differentiation process (tdTomato^+^; orange). The direct differentiation pathway preferentially produced niche-supporting MKs while the stepwise process produced immune-associated MKs, both processes equally produced platelet-producing MKs. The direct differentiation pathway was increased upon myeloablation (B; a single dose of chemotherapy agent 5-fluorouracil) while inflammation increased the stepwise differentiation pathway (C; a single dose of lipopolysaccharide). HSPC = hematopoietic stem and progenitor cell, MK = megakaryocyte, MkP = megakaryocyte progenitor.

Murine lineage-tracing models have been prominent tools for understanding hematopoiesis.^2,5,6^ Li et al^5^ generated *Cd48*^dre^*;R26*^*rox-tdTomato*^ mice by introducing a constitutively active Dre recombinase behind the endogenous *Cd48* gene stop codon and crossed them with *Rosa26*^*rox-STOP-rox-tdTomato*^ *(R26*^*rox-tdTomato*^) mice. Flow cytometry analyses of 8-week-old mice confirmed that, in this model, hematopoietic stem cells (HSCs; defined as lineage negative, c-Kit^+^Sca-1^+^ [LKS^+^] CD34^−^CD135^−^) and multipotent progenitors (MPPs; LKS^+^CD34^+^CD135^−^) were tdTomato^−^. The majority of downstream progenitors were tdTomato^+^, with the exception of MkPs and MKs, which comprised approximately 50% tdTomato^−^ cells, indicating that they emerged from the direct differentiation pathway^5^ (**Fig. 1A**).

To generate a model to fate map *Cd48*-Dre-targeted cells, Li et al^5^ generated the tamoxifen-inducible *Ubc*-creER*;Cd48*^*dre*^*;R26*^*ZT1*^ mice, which labeled *Cd48*-targeted lineages with tdTomato and lineages generated from cells that never expressed *Cd48* with ZsGreen at the time of tamoxifen induction.^5^ Li et al^5^ injected tamoxifen intraperitoneally into 2-month-old *Ubc-creER;Cd48*^*dre*^*;R26*^*ZT1*^ mice for 3 consecutive days and analyzed them 1 day later. Flow cytometry revealed that 80% to 90% of HSCs and MPPs expressed ZsGreen, and 80% to 90% of CMPs, GMPs, MEPs, common lymphoid progenitors (CLPs), myeloid, erythroid, B and T cells were labeled with tdTomato. There were equal proportions of MKs that expressed tdTomato or ZsGreen.^5^

Longer term analyses of the *Ubc*-creER*;Cd48*^*dre*^*;R26*^*ZT1*^ mice provided valuable insight into the turnover cycles of each of the *Cd48*-targeted hematopoietic cell populations, because the tdTomato^+^ cells were replaced by ZsGreen cells. This revealed that it took approximately 30 days for *Cd48*-targeted tdTomato^+^ MKs to disappear in the mice, with the majority being depleted between 5 and 14 days post-tamoxifen. Similar observations were obtained using *Kit*-creER in place of *Ubc*-creER.^5^

Li et al^5^ then performed scRNA-seq on CD41^+^ MKs isolated from the bone marrow of *Cd48*^*dre*^*;R26*^*rox-tdTomato*^ mice. Differential gene expression analysis defined four MK clusters that were termed platelet-producing MKs, cycling MKs, niche-supporting MKs and immune MKs, consistent with their previous report.^7^ The *Cd48*-targeted tdTomato^+^ cells (generated by stepwise differentiation) contained most of the immune MKs (*Cd53* and *Lsp1* high) but few niche-supporting MKs; and *Cd48*^−^ tdTomato^−^ cells (derived by direct differentiation) contained most of the niche-supporting MKs (*Igf1* and *Mylk4* high) but few immune MKs.^5^ The platelet-producing and cycling MKs were distributed equally between both groups (**Fig. 1A**). In comparison to *Cd48*^−^ tdTomato^−^ MKs, the *Cd48*-targeted tdTomato^+^ MKs contained more 2N and fewer 32N MKs and had 10-fold greater phagocytic activity, a feature of immune MKs.^7,8^ The authors also found that 13% of *Cd48*-targeted tdTomato^+^ MKs expressed major histocompatibility complex (MHCII) compared to 1% of *Cd48*^−^ tdTomato^−^ MKs. Furthermore, confocal imaging of bone marrow sections showed *Cd48*^−^ tdTomato^−^ MKs localized closer to *Ctnnal1*^GFP^ HSCs.^5^

The 2 MK differentiation pathways responded differently to physiological stressors. There was an increase in the frequency of *Cd48*^−^ tdTomato^−^ MKs produced by the direct differentiation pathway in response to myeloablation by 5-fluorouracil (5-FU) (**Fig. 1B**). In contrast, *Cd48*-targeted tdTomato^+^ MKs produced by stepwise differentiation were increased after lipopolysaccharide (LPS)-induced inflammation (**Fig. 1C**). Notably, scRNA-seq studies revealed that the niche-supporting MK cluster obtained from 5-FU–treated mice (8 days post-5-FU) increased from 12% to 27% compared to the control. Furthermore, the immune MK cluster obtained from the LPS-treated mice (12 hours post-treatment) increased from 34% to 50% compared to the control.

Collectively, the studies by Li et al^5^ have provided additional evidence that megakaryopoiesis is complex, involving distinct differentiation pathways that produce functionally different MKs with diverse physiological roles. Using murine lineage-tracing models, the authors showcased the kinetics and functional differences between the stepwise and direct pathways.

It is worth noting that the *Itga2b* (CD41) expression level in the immune cluster was lower than in the other three subpopulations. If this is also observed for cell surface expression, CD41 expression levels can potentially be used to separate the immune MKs from the other MK subpopulations.

Surprisingly, there were very few *Cd48*^*+*^ tdTomato^+^ cells observed in the HSC (LKS^+^CD34^−^CD135^−^) and MPP (LKS^+^CD34^+^CD135^−^) populations. It has previously been shown that the MPP population contains significant proportions of both MPP2 (LKS^+^CD135^−^CD150^+^CD48^+^) and MPP3 (LKS^+^CD135^−^CD150^−^CD48^+^), cells that express high levels of CD48.^9^ This discrepancy is likely due to a difference in gating strategies used to identify the cells in the current study and it would be beneficial to revise the flow cytometry gating strategies to be consistent with those validated by previous publications. This may also explain some discrepancies to the studies of platelet production in young FlkSwitch reporter mice (*Cd135*^*Cre*^*;R26*^*mTmG*^) generated by the Forsberg group.^2^ In the FlkSwitch reporter mice, the majority of MkPs and platelets in young mice expressed GFP, indicating that they were generated via *Cd135*-expressing HSPCs (the majority which co-express CD48^9^). Intriguingly, a recent study using the FlkSwitch reporter mice identified a distinct subpopulation of *Cd135*^−^ tdTomato^+^ MkPs in aged (24-month-old) mice and showed that the *Cd135*^−^ tdTomato+ platelets were hyper-reactive.^10^ It would be worth determining if the *Cd135*^−^ tdTomato^+^ aged MkPs preferentially produce the MK subpopulations identified in the *Cd48*^*dre*^ mice.^5^ Furthermore, it would be of interest to determine if the platelets produced by *Cd48*^−^ tdTomato^−^ MKs are hyper-reactive in both young and aged mice.

Additionally, the 2N CD41^+^ MKs isolated in the current study would include CD41^+^ HSCs^6^ and other cells that express CD41, including MkPs, and it is important to exclude those cells in future studies. Irrespective, the findings reported by Li et al^5^ improve our understanding of megakaryopoiesis and provide novel mouse models that will be very useful for further studies of hematopoiesis, including megakaryopoiesis.

## References

[1] Noetzli LJ French SL Machlus KR. New insights into the differentiation of megakaryocytes from hematopoietic progenitors. Arterioscler Thromb Vasc Biol 2019;39(7):1288–1300. 31043076 10.1161/ATVBAHA.119.312129 PMC6594866

[2] Boyer SW Schroeder AV Smith-Berdan S Forsberg EC. All hematopoietic cells develop from hematopoietic stem cells through Flk2/Flt3-positive progenitor cells. Cell Stem Cell 2011;9(1):64–73. 21726834 10.1016/j.stem.2011.04.021 PMC4103692

[3] Sanjuan-Pla A Macaulay IC Jensen CT. Platelet-biased stem cells reside at the apex of the haematopoietic stem-cell hierarchy. Nature 2013;502(7470):232–236. 23934107 10.1038/nature12495

[4] Yamamoto R Morita Y Ooehara J. Clonal analysis unveils self-renewing lineage-restricted progenitors generated directly from hematopoietic stem cells. Cell 2013;154(5):1112–1126. 23993099 10.1016/j.cell.2013.08.007

[5] Li JJ Cheng H Cheng T. Differentiation route determines the functional outputs of adult megakaryopoiesis. Immunity 2024;57(3):478–494.e6. 38447571 10.1016/j.immuni.2024.02.006

[6] Gekas C Graf T. CD41 expression marks myeloid-biased adult hematopoietic stem cells and increases with age. Blood 2013;121(22):4463–4472. 23564910 10.1182/blood-2012-09-457929

[7] Sun S Jin C Si J. Single-cell analysis of ploidy and the transcriptome reveals functional and spatial divergency in murine megakaryopoiesis. Blood 2021;138(14):1211–1224. 34115843 10.1182/blood.2021010697 PMC8499048

[8] Pariser DN Hilt ZT Ture SK. Lung megakaryocytes are immune modulatory cells. J Clin Invest 2021;131(1):e137377. 33079726 10.1172/JCI137377 PMC7773372

[9] Pietras EM Reynaud D Kang Y-A. Functionally distinct subsets of lineage-biased multipotent progenitors control blood production in normal and regenerative conditions. Cell Stem Cell 2015;17(1):35–46. 26095048 10.1016/j.stem.2015.05.003 PMC4542150

[10] Poscablo DM Worthington AK Smith-Berdan S. An age-progressive platelet differentiation path from hematopoietic stem cells causes exacerbated thrombosis. Cell. Cell 2024;187(12):3090–3107.e21. 38749423 10.1016/j.cell.2024.04.018 PMC12047039

